# Influence of protocol scan on choroidal vascularity measurements: a spectralis optical coherence tomography study

**DOI:** 10.1038/s41433-022-02255-4

**Published:** 2022-09-28

**Authors:** Claudio Iovino, Paolo Melillo, Paolo Capriuoli, Kiran Kumar Vupparaboina, Francesco Testa, Jay Chhablani, Francesca Simonelli

**Affiliations:** 1 Eye Clinic, Multidisciplinary Department of Medical, Surgical and Dental Sciences, University of Campania Luigi Vanvitelli, Naples, Italy; 2 UPMC Eye Centre, University of Pittsburgh, Pittsburgh, PA USA

**Keywords:** Medical research, Outcomes research

## Abstract

**Objectives:**

To compare choroidal vascularity index (CVI) measurements using the automated image binarization algorithm in healthy subjects with two Spectralis spectral-domain optical coherence tomography (SD-OCT) protocol scans.

**Methods:**

Sixty-nine eyes of 69 healthy volunteers were included in this cross-sectional prospective study. Two subsequent horizontal 20°line scans passing through the fovea were acquired with enhanced-depth imaging mode with high speed (HS) and high resolution (HR) protocol scans. CVI and its subcomponents were measured with the previously validated automated algorithm. Differences between choroidal measurements obtained with HS and HR protocol scans were evaluated with t-test and Bland & Altman plots.

**Results:**

A total of 33 male (47.8%) and 36 female (52.2%) subjects with a mean age of 35.1 ± 13.4 years were included. Overall, HS protocol scan was associated with significant lower values of total choroidal area (−0.047 mm^2^) and stromal choroidal area (−0.036 mm^2^), and a significant greater value of CVI (+0.010%) if compared to HR protocol. Luminal choroidal area was lower when calculated with the HS protocol, although it did not reach significance. To compare the two different protocols, the number of pixels should be multiplied for 3.87 ×5.73 when the CVI is measured on a HR OCT b scan and 3.87 ×11.46 for the HS OCT b scan.

**Conclusions:**

HS and HR acquisition modes significantly influence CVI and its subcomponents values measured with the automated software. However, adopting the scale factors can minimize the differences between the two protocol scans.

## Introduction

The choroid is one of the most vascularized tissue of the body and contributes to the majority of oxygen and other nutrients supply to the outer retina and the retinal pigment epithelium (RPE) [1]. It is composed of three vascular layers: Haller’s layer with large-sized vessels, Sattler’s layer with medium-sized vessels and choriocapillaris. Vascular changes of the choroid play an important role in the pathogenesis of many retinal diseases [1].

The advent of the optical coherence tomography (OCT) over the last decades has allowed a detailed and non-invasive examination of the choroidal vessels [2]. Particularly, the introduction of enhanced depth imaging (EDI) spectral domain (SD)-OCT and swept source (SS)-OCT have provided a more in-depth analysis of the choroidal structure. Although choroidal thickness (CT) is considered a robust tool in clinical research, it only reflects the total choroidal structure including stroma and vasculature [2, 3].

Conversely, the new OCT metric named choroidal vascularity index (CVI) allows to calculate the area of dark and light pixels corresponding to the luminal and stromal areas of the choroid [4, 5]. CVI, a ratio of the luminal choroidal area (LCA) and total choroidal area (TCA), was originally calculated through a semi-automated algorithm requiring a manual selection of the TCA followed by the application of several auto-local thresholds to binarize the image and select dark pixels [6–9].

More recently, fully automated choroidal segmentation and binarization algorithms allowing a significant reduction of the time required for the analysis have been reported [10–15]. Several authors investigated the differences in terms of CVI values measured with SD-OCT or SS-OCT [16], or between the different algorithms employed [17]. Nevertheless, the influence of different OCT protocol scans on automatized CVI measurement has never been studied.

The two Spectralis protocol scans, high resolution (HR) and high speed (HS), differ in terms of resolution and speed of scan acquisition.

In this study, we compared CVI measurements and its subcomponents using the automated image binarization algorithm in healthy subjects with both the HS and HR Spectralis protocol scans.

## Methods

This was a prospective, cross-sectional study conducted at Referral Center of Hereditary Retinal Dystrophies of the University of Campania “Luigi Vanvitelli”. The investigation was approved by the Local Ethical Committee of the University of Campania “Luigi Vanvitelli” and performed according to the guidelines of the Declaration of Helsinki. After receiving a detailed explanation of the study, written informed consent was obtained from all participants before examination.

### Patients and clinical examination

All subjects were screened for the presence of any ocular disease through a complete ophthalmologic examination including best corrected visual acuity (BCVA), intraocular pressure, slit-lamp biomicroscopy and fundus examination.

Exclusion criteria were patients younger than 18 or older than 70 years, ocular surgery in the previous 6 months, history of any systemic and ophthalmic disease, pregnancy, and a spherical equivalent refractive error greater than −6 D or +3 D. In addition, patients with media opacities that could influence image quality were also excluded from the study. Particularly, all scans with poorly visible choroidal scleral interface were excluded. All individuals required a BCVA of 20/25 or better.

### Image acquisition

All patients were imaged using the Spectralis SD-OCT version 6.12.3.0 (Spectralis, Heidelberg Engineering, Heidelberg, Germany). Two subsequent horizontal 20°line scans passing through the fovea were acquired with EDI mode in HS and HR. Patients were instructed to see the central blue light and not blink the eye during the examination. All OCT examinations were performed between 10:00 and 12:00 am. The operator (P.C.) was trained to the study protocol and performed all OCT scans.

The CVI was calculated using the previously reported automated algorithm, that included initial denoising with localization of the choroidal inner and outer boundary [10, 12, 13]. To allow computation of LCA and stromal choroidal area (SCA), the OCT B-scan was binarized and choroidal components were segmented. Automated binarization process included exponential and non-linear enhancement, and thresholding. The dark regions were labeled as LCA and the bright regions as SCA. TCA was measured as the sum of the SCA and LCA, and the CVI was calculated as the ratio of LCA over TCA. All choroidal measurements were performed for both HS and HR Spectralis protocol scans. To compare the measurement of CVI subcomponents obtained with the two protocol scans, the area of LCA and TCA were estimated in µm^2^, by multiplying the number of pixel per scaling Z (i.e., 3.87 µm/pixel for both protocol scans) and per scaling X factor (i.e., 11.46 for HS; 5.73 for HR protocol scan).

### Statistical analysis

Continuous variables are reported as mean ± standard deviation (SD) and categorical variables are reported as counts (frequency). Differences between measurements obtained with HS and HR protocol scans were evaluated with *t*-test and Bland & Altman plots. *P*-values lower than 0.05 were considered as statistically significant.

## Results

A total of 69 eyes of 69 healthy volunteers were included in the study. Only the right eye was selected for the analysis. There were 33 male (47.8%) and 36 female (52.2%) subjects with a mean age of 35.1 ± 13.4 years.

Regarding choroidal measurements, mean TCA was 1.86 ± 0.52 mm^2^ and 1.91 ± 0.54 mm^2^ measured with HS and HR protocol scan respectively (*p* = 0.005).

Mean SCA and LCA were 0.65 ± 0.21 mm^2^ vs 0.69 ± 0.22 mm^2^ (*p* = 0.0004) and 1.21 ± 0.32 mm^2^ vs 1.22 ± 0.33 mm^2^ (*p* = 0.255) for HS and HR protocols, respectively. Regarding CVI evaluation, we found a mean of 0.65% ± 0.04% with HS protocol scan and 0.64% ± 0.03% with HR (*p* = 0.001).

**Fig. 1:**
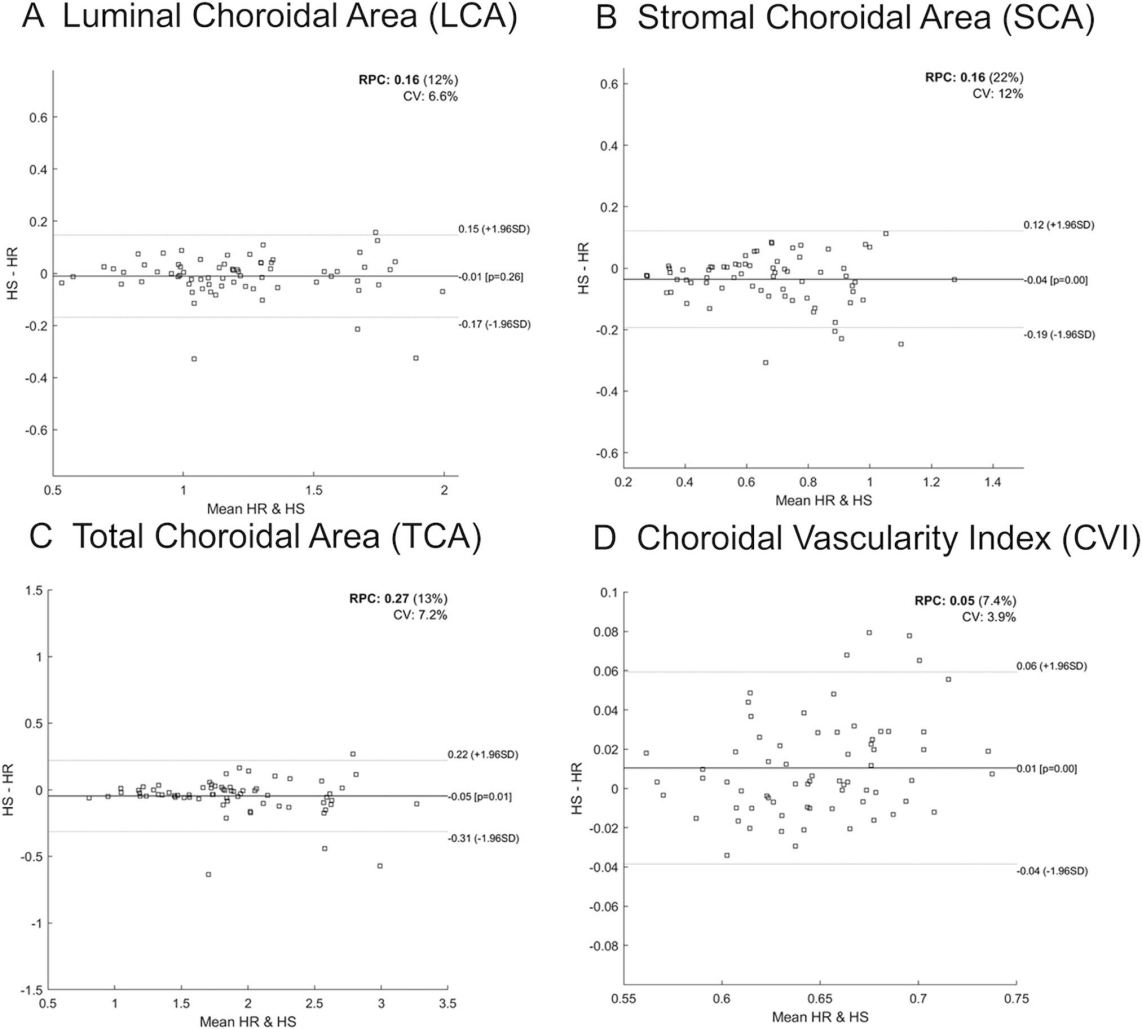
Bland–Altman plots comparing the choroidal measurements between high resolution (HR) and high-speed (HS) spectral-domain optical coherence tomography scan protocols. The black lines indicate the mean difference between HR and HS, whereas the gray dotted lines denote the 95% confidence intervals. Specifically, Bland–Altman plot shows no significant difference in LCA measurements between HS and HR protocols (**A**); lower values of SCA (**B**) and of TCA (**C**) with HS protocol compared to HR; greater value of CVI (**D**) with HS protocol compared to HR.

Overall, as shown in Fig. 1, HS protocol scan gave significant lower values of TCA (−0.047 mm^2^) and SCA (−0.036 mm^2^), and a significant greater value of CVI (+0.010%) if compared to HR protocol.

**Fig. 2:**
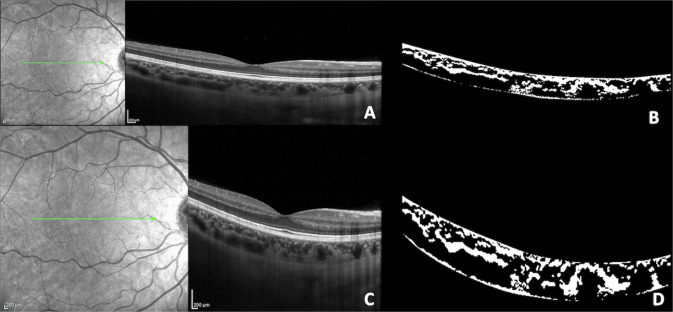
Spectral-domain optical coherence tomography (OCT) B scans (using enhanced-depth imaging mode) and corresponding binarized OCT scans from a 38-year-old healthy man acquired with High Resolution (HR) and High Speed (HS) mode. Registered near-infrared reflectance images are also included to validate the same scan position for both protocol scans. **A**, **B** OCT B scan and corresponding binarized scan acquired with HR mode. The total choroidal area (TCA) measured 2.96 mm^2^, the stromal choroidal area (SCA) measured 0.98 mm^2^, the luminal choroidal area (LCA) measured 1.97 mm^2^, and the choroidal vascularity index (CVI) measured 66.7%. **C**, **D** OCT B scan and corresponding binarized scan acquired with HS mode. The TCA, SCA, and LCA measured 2.84 mm^2^, 0.88 mm^2^, and 1.96 mm^2^, respectively. The CVI measured 68.7%.

To compare the two different protocols, the number of pixels should be multiplied for 3.87 ×5.73 when the CVI is measured on a HR OCT b scan and 3.87 ×11.46 for the HS OCT b scan. A representative case of CVI measurements and its subcomponents measured with the two acquisition modes is shown in Fig. 2.

## Discussion

CVI has been widely recognized as a valuable, repeatable and robust OCT metric for both healthy and disease eyes [12, 15, 18]. The automated software employed to measure CVI provides the capability to calculate quantitative parameters of the choroid and stratify the vascular and stromal components [4]. Several authors investigated the differences in terms of CVI values measured with SD-OCT or SS-OCT[16], or between different algorithms [17] and different scanning area [19], reporting a high reproducibility in all cases [16, 17, 19].

More recently, Esroz et al. investigated the repeatability of CVI values in centered and decentered directional subfoveal OCT scans [20]. The authors found a moderate agreement between CVIs obtained from scans acquired using a different pupil entry position of the beam and suggested to always use the same direction of the beam to standardize CVI measurements [20]. Corvi et al., analyzed the repeatability of OCT angiography derived retinal quantitative metrics using HR versus HS acquisition modes, reporting statistically significant different values [21].

In the present study, we compared CVI measurements and its subcomponents using the automated image binarization algorithm in healthy subjects with both the HS and HR Spectralis protocol modes. The two protocols differ with regard of the resolution of the B-scan with twice as many pixels of acquisition with the HR mode. Higher resolution and greater number of pixels for acquisitions require longer scan times. The scaling factor adopted from the automatized software used for CVI evaluation has been originally set on the HS protocol scan [10, 12, 13]. Particularly, there are two different resolutions along the X axis for the two protocol scans: 5.73 μm/pixel for HR and 11.46 μm/pixel for HS resolution. On the other hand, the resolution along the vertical axis is the same (3.87 μm/pixels). These different resolutions influence the analysis of the CVI and its subcomponents. As demonstrated, HS protocol scan is associated with a significant greater value of CVI and significant lower values of TCA and SCA if compared to HR protocol. In absolute numbers, being the X axis 11.46 μm/pixel for HS resolution and 5.73 μm/pixel for HR, the TCA and SCA were both significantly lower with HS protocol and consequently the CVI was significantly higher. Regarding LCA, it was also lower when calculated with the HS protocol although it did not reach significance.

Of note, considering that once the OCT b scan has been acquired with the HS or HR protocol scan it is not possible to change it back to the other one, a conversion factor to allow comparisons between different protocols resolution could be very useful for retrospective studies. To compare the two different protocols, the number of pixels should be multiplied for 3.87 ×5.73 when the CVI is measured on a HR OCT b scan and 3.87 ×11.46 for the HS. By adopting these proportions, OCT scans acquired with the two different protocols can be compared with limited bias of the results. This is of particular importance in case-control or cohort studies where the CVI or its subcomponents can be compared even with B scan acquired with either HR and HS mode. CVI analysis remains in a state of rapid evolution and development, and in a near future it could be embedded in the software of all OCT machines, providing additional information about the vascular status of the choroid.

Of note, the automated software allows a significant reduction in the time required for a precise quantitative analysis of the choroidal vasculature and therefore its use is increasing among researchers worldwide.

Moreover, all the considerations made for the choroidal vasculature measurements performed with the automated software should be also taken into account for the semiautomated analysis originally proposed by Agrawal and coauthors [22]. Particularly, all ophthalmologists/technicians should be aware of the different pixel proportions on the X axis when calculating CVI with the semiautomated software as well. The Heidelberg Spectralis is the only OCT allowing the two HR and HS acquisition modalities, but it would be interesting to also investigate the variability in the CVI evaluation among several OCT machines to ascertain any discrepancies in terms of pixel resolution influencing the CVI and its subcomponents values.

Limitations of the present analysis include the relatively small number of subjects included. As a result, the study could be underpowered to detect very small differences in the various choroidal OCT metric evaluations, although only LCA did not reach significance. Additionally, the follow-up function was not applied when performing OCT scans, but this is not allowed from the manufacture when using different protocol scans.

In summary, we observed that HS and HR acquisition modes for Spectralis OCT influence the CVI and its subcomponents values measured with the automated software. However, adopting the proposed scale factors can minimize the differences between the two acquisition modes. Considering the significant interest in using the CVI in the clinical setting, the results of our study highlight an important technical issue never reported before.

### Summary

#### What was known before

- Automatic algorithms allow a significant reduction of the time required for the analysis of the choroidal vascularity index (CVI).

#### What this study adds

- High speed and high resolution acquisition modes for Spectralis optical coherence tomography influence the CVI and its subcomponents values measured with the automated software.
- Adopting the proposed scale factors can minimize the differences between the two scan protocols.

## Data Availability

The dataset generated during and/or analyzed during the current study are available from the corresponding author on reasonable request.
